# A Negatively Curved Pyrene‐Fused Azaacene

**DOI:** 10.1002/anie.3918437

**Published:** 2026-04-29

**Authors:** Marco Carini, Miguel Martín‐Arroyo, Manuel Melle‐Franco, Aurelio Mateo‐Alonso

**Affiliations:** ^1^ POLYMAT, Department of Applied Chemistry University of the Basque Country (EHU) Donostia‐San Sebastián Spain; ^2^ CICECO—Aveiro Institute of Materials Department of Chemistry University of Aveiro Aveiro Portugal; ^3^ Ikerbasque, Basque Foundation For Science Bilbao Spain

**Keywords:** curved aromatics, overcrowding, polycyclic aromatic hydrocarbons

## Abstract

Non‐planar aromatic hydrocarbons display distorted π‐frameworks that give rise to unique optoelectronic properties. Among the different strategies for generating non‐planar aromatic hydrocarbons, steric overcrowding has afforded numerous twisted structures displaying helical or alternate twists, whereas bent structures remain rare. Herein, we report a pyrene‐fused azaacene derivative in which eight strategically positioned phenyl substituents enforce bending of the aromatic core rather than twisting, generating a negatively curved, saddle‐shaped structure. Single‐crystal x‐ray diffraction reveals large deviations from planarity with bend angles of 41° and 34°, stabilised by intramolecular π‐π stacking between facing phenyl rings.

## Graphical abstract

A pyrene‐fused azaacene derivative is reported in which steric overcrowding caused by eight phenyl substituents induces bending of the aromatic framework rather than twisting.

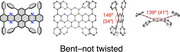

Non‐planar aromatics are polycyclic aromatic hydrocarbons in which the aromatic framework deviates from planarity as a result of steric congestion, strain, or topological constraints [1, 2, 3, 4, 5, 6, 7, 8, 9, 10, 11, 12, 13, 14]. These deviations can reshape conjugation and chirality, enabling optoelectronic, chiroptical, spintronic, and molecular recognition properties not present in flat systems. Another important aspect is the enhanced solubility typically observed in non‐planar aromatics, arising from their lower tendency to aggregate through π‐stacking, which makes their purification, characterization and processing easier. Non‐planar aromatics are generally obtained through twisting or bending, giving rise to twisted and bent (or curved) aromatics.

On one hand, twisted aromatics arise from distortion of the π‐framework along its longitudinal axis, either with twists in the same direction (helical) or in opposite directions (alternated) (Figure 1a,b). Twisted aromatics are typically obtained by substituent overcrowding (e.g., twistacenes and related systems [2, 6, 7, 15, 16, 17, 18]), angular fusion (e.g., helicenes and related systems) [4, 6, 19, 20, 21], the incorporation of non‐hexagonal rings [22, 23], and macrocyclic or topological closures (e.g., planar chiral cyclophanes [24, 25, 26, 27] and Möbius belts [28, 29]).

**FIGURE 1:**
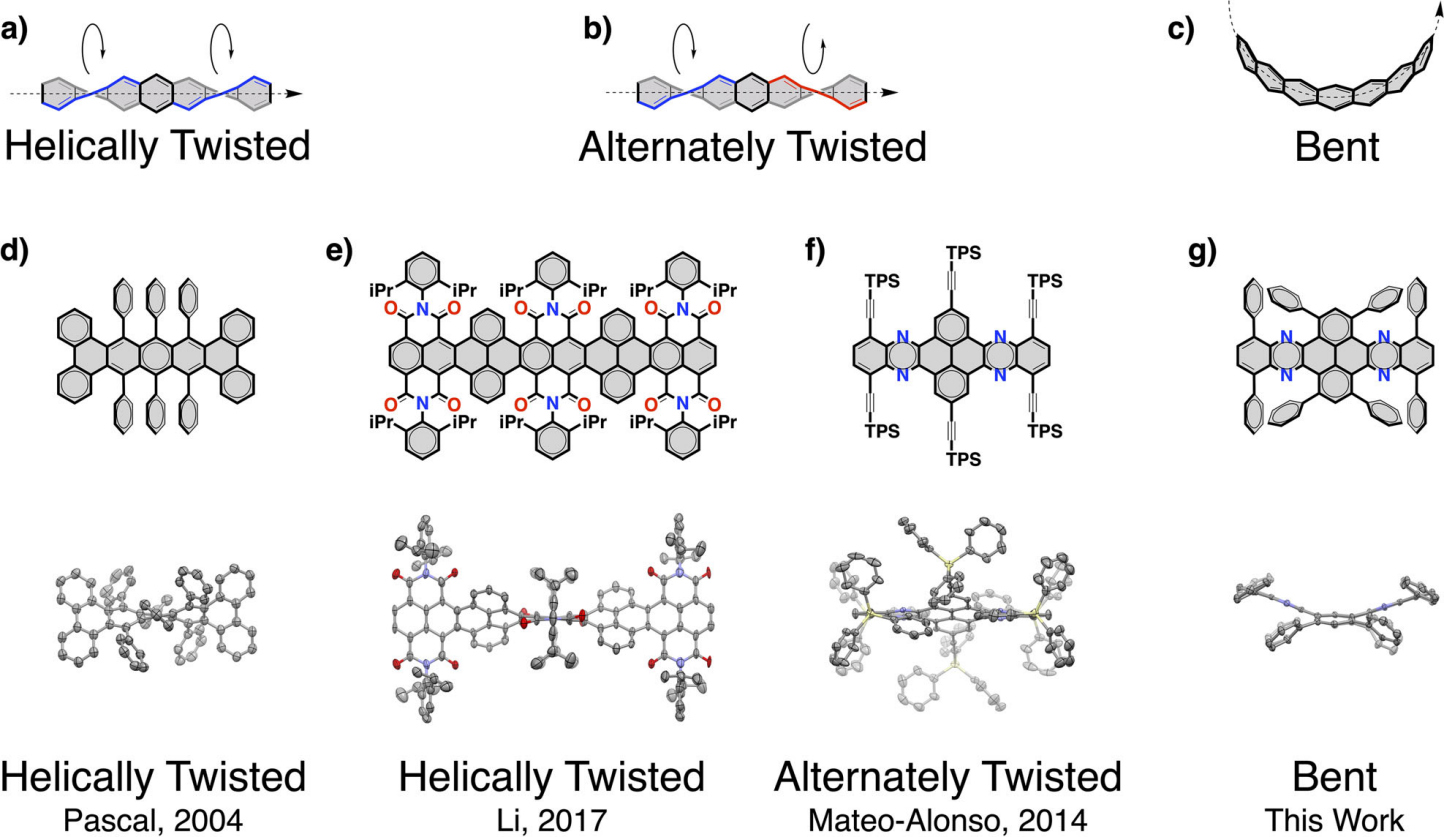
General structures depicting (a) helically twisted, (b) alternately twisted, and (c) bent molecules. (d–f) Representative examples of twistacene derivatives [42, 43, 44] obtained by substituent overcrowding and the (g) structure of bent pyrene‐fused azaacene **1** described in this work.

On the other hand, bent (or curved) aromatics result from deformation of the π‐framework perpendicular to its plane, producing structures (Figure 1c) with positive (concave or convex) or negative (concave and convex) curvature. Bent aromatics have typically been obtained through geometric or topological constraints, such as macrocyclic or topological closures (e.g., end‐to‐end strapped cyclophanes [5, 30, 31, 32], carbon nanobelts [32]), the incorporation of non‐hexagonal rings [8, 9, 10, 14], ring strain [6, 33, 34], or angular fusion [35, 36, 37, 38, 39].

Bent aromatics are generally less accessible than their twisted relatives [40, 41], a contrast well illustrated by substituent overcrowding, in which steric congestion is generally relieved through torsional distortion rather than by out‐of‐plane bending. In fact, while substituent overcrowding has afforded numerous twisted structures with helical or alternate twists [2, 6, 7, 42, 43, 44, 45, 46] (representative examples [42, 43, 44] are shown in Figure 1d–f), bent structures remain rare [36, 47, 48, 49, 50, 51, 52]. This predominance of twisted over bent structures is even more evident in pyrene‐fused azaacenes, where nitrogen substitution reduces steric congestion in the bay region by removing the confronting bay‐site groups and by keeping the substituents farther apart, so that only planar or alternately twisted structures have been observed (Figure 1f), even when highly sterically demanding groups are introduced [7, 44, 45, 46, 53, 54, 55, 56, 57, 58, 59, 60].

Herein, we describe a pyrene‐fused azaacene (**1**) in which steric overcrowding, introduced by eight phenyl substituents at key positions, induces pronounced bending of the aromatic framework rather than twisting (Figure 1g). This deformation generates a negatively curved, saddle‐like structure that exhibits significant deviations from planarity, with bend angles of 41° and 34° as determined by single‐crystal x‐ray diffraction. The stabilization of such a bent conformation is consistent with intramolecular π‐stacking between facing pairs of phenyl substituents and with theoretical calculations, which also predict the bent conformation to be the most stable.

The synthesis of dibenzohexacene **1** relies on the condensation of 1,3,6,8‐tetraphenylpyrene‐4,5,9,10‐tetraone (**2**) with 1,4‐diphenyl‐3,4‐diaminobenzene (Scheme 1). Pyrenetetraone **2** was synthesised from pyrene in three steps. Pyrene was first tetrabrominated to obtain 1,3,6,8‐tetrabromopyrene, which was then subjected to a Suzuki coupling with phenylboronic acid to afford 1,3,6,8‐tetraphenylpyrene **3**, following reported procedures (Scheme S1) [61, 62, 63]. The oxidation of tetraphenylpyrene **3** was initially attempted under the conditions reported by Harris for unsubstituted pyrene [64], namely NaIO_4_ in the presence of RuCl_3_ in a dichloromethane/acetonitrile/water mixture at 40°C. However, these conditions did not afford the desired product. By replacing dichloromethane with carbon tetrachloride, the reaction temperature could be increased to 60°C, enabling successful oxidation of tetraphenylpyrene **3** to tetraone **2** in 17% yield. Then, 1,4‐diphenyl‐3,4‐diaminobenzene was synthesised in three steps following reported procedures (Scheme S1) [65, 66]. Finally, tetraphenylpyrenetetraone **3** was then cyclocondensed with 1,4‐diphenyl‐3,4‐diaminobenzene in refluxing acetic acid, affording dibenzohexacene **1** in 69% yield after recrystallisation.

**SCHEME 1:**
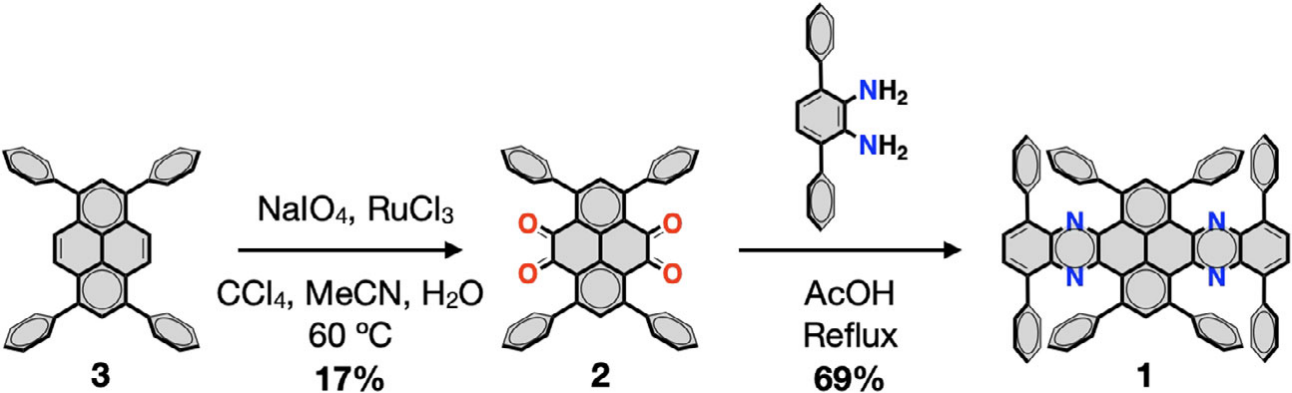
Synthesis of dibenzohexacene **1**.

Single crystals of dibenzohexacene **1** suitable for x‐ray analysis were obtained by vapor diffusion of ethanol into a 1,1,2,2‐tetrachloroethane solution [67]. Dibenzohexacene **1** is strongly distorted and adopts a saddle‐shaped conformation in which the quinoxaline units formed by rings A/B and E/F bend in the opposite direction to the curvature defined by the G/H ring pair (Figure 2b). This negatively curved structure exhibits deviations from planarity of 41° and 34° at the intersections between the planes defined by the B/E and G/H rings, respectively. The phenyl substituents on the pyrene and quinoxaline segments adopt average dihedral angles of 53° and 47°, respectively. Notably, the phenyl substituents on the quinoxaline segments are positioned above those on the pyrene segments in a nearly parallel fashion at an average separation of 3.6 Å, consistent with intramolecular π‐π stacking that stabilises the saddle negatively‐curved conformation. This bent structure contrasts with previous reports on dibenzotetraazahexacene derivatives, which have exclusively shown alternately twisted conformations [7, 44, 45, 46]. Dibenzohexacene **1** forms 1:3 co‐crystals with the solvent 1,1,2,2‐tetrachloroethane (not shown). A concave‐to‐concave packing motif is observed, in which the ABC rings of one molecule stack against the DEF rings of a neighbouring molecule at 3.9 Å.

**FIGURE 2:**
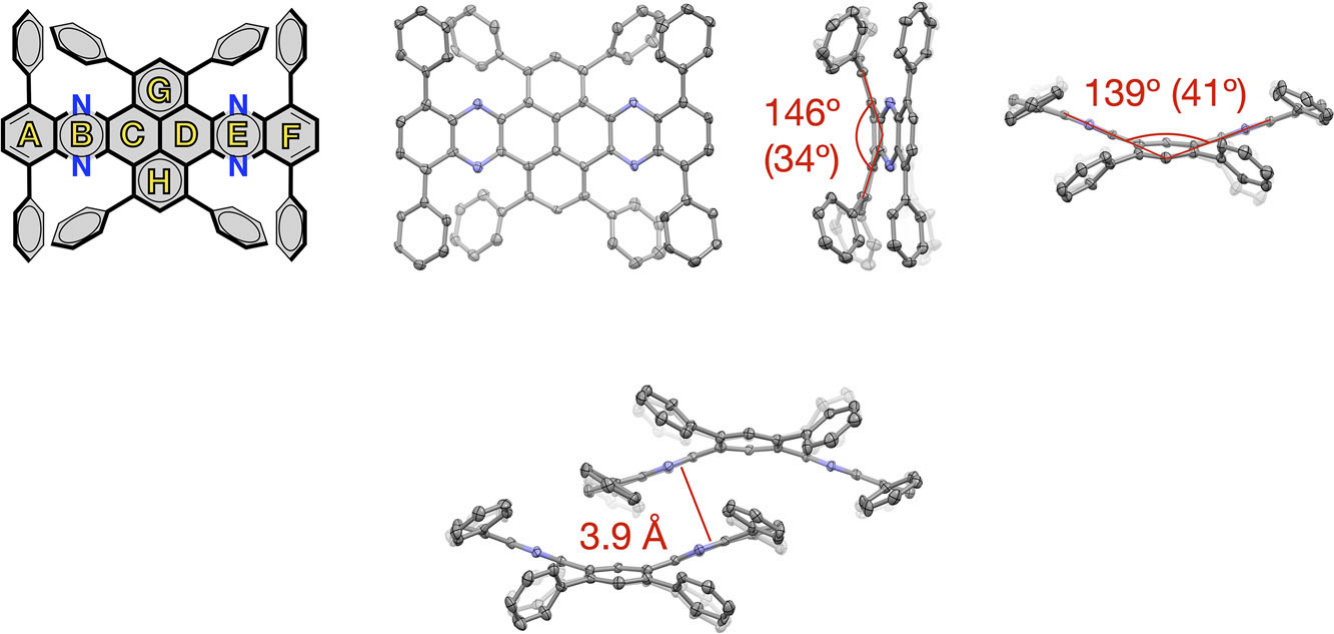
Different views of the single crystal x‐ray structures of dibenzohexacene of **1**. The ring lettering indicates the rings discussed in the main text.

To understand the preferential formation of the bent structures, we carried out a conformational exploration of dibenzohexacene **1**. An exhaustive search was performed within the Conformer‐Rotamer Ensemble Sampling Tool (CREST) [68], based on a Semiempirical Quantum Mechanics (SQM) Hamiltonian [69]. The so‐obtained conformations were then refined and ranked with DFT at the M06‐2X‐6‐31G(d,p) level first. The most likely conformations were then refined and ranked with Grimme's ωB97X‐3c Hamiltonian, chosen for its remarkable accuracy and computational efficiency [70]. These calculations were performed at the gas phase and within the SMD model of dichloromethane continuum (Table S1). Harmonic frequencies were computed to check the effect of thermodynamic vibrational thermal corrections, as well as to confirm that the found conformations were all minima. Compound **1**, which contains eight phenyl groups, exhibits a rich conformational landscape, giving rise to several accessible conformations in dichloromethane, including *bent* (94.3%), *asymmetrically bent* (4.2%), *bent‐twisted* (0.9%), *helically twisted* (0.5%), and *alternately twisted* (0.1%) conformations, among which, the *bent* conformation found in the crystal structure is by far the most populated one (Figure 3 and Table S1). The calculations indicate that the non‐planar structures are the results of steric hindrance that are further stabilized by π‐π stacking between the phenyl substituents in the pyrene residue and those in the pyrazino‐quinoxaline residues. An additional stabilization may also arise from an n‐π interaction between the nitrogens’ lone pairs and the phenyl groups in the pyrene residues. The preferential stabilization of the *bent* versus the *twisted* conformations can be explained in terms of the more efficient π overlap between the phenyl groups in the pyrene residue and those in the pyrazino‐quinoxaline residues. We attempted to estimate the inversion barriers by variable‐temperature NMR experiments. Although chemical‐shift changes and partial signal coalescence were observed for some signals, these features cannot be unambiguously attributed to the inversion process, as they may also arise from rotation of the peripheral phenyl rings (Figure S1).

**FIGURE 3:**
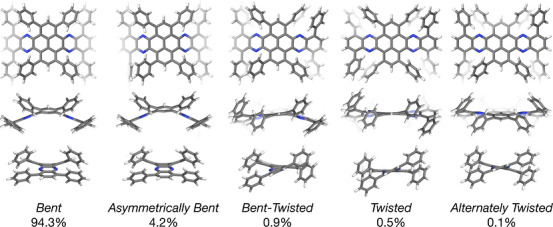
Most likely conformations of dibenzohexacene **1** computed with the ωB97X‐3c Hamiltonian.

The optoelectronic and redox properties were investigated by steady‐state absorption and fluorescence spectroscopies and cyclic voltammetry. Dibenzohexacene **1** is a bright yellow solid with a faint blue fluorescence in solution. The UV‐vis absorption spectra of **1** (Figure 4a) show the typical ρ and ß bands observed in similar dibenzohexacene derivatives [44, 45, 46]. While similar dibenzotetraazahexacenes with acetylene and silyl substituents are generally fluorescent, a very weak fluorescence (Φ = 0.02%) was detected for dibenzohexacene **1** (Figure 4a). A likely rationale for the low quantum yield is the bent nature of the core, consistent with previous observations on pyrenophanes [71] and anthracenophanes [27, 72], which show that the quantum yield decreases as distortion of the π‐system increases. The cyclic voltammograms of dibenzohexacene **1** show a first reversible reduction and two consecutive irreversible reductions (*E*(V) = −1.81 (half‐wave), −2.04 (cathodic peak), −2.29 (cathodic peak), and −2.43 V (cathodic peak)) and no oxidation processes within the solvent‐supported electrolyte window (Figure 4b). A HOMO‐LUMO gap of 2.63 eV was estimated from the onset of the lowest energy absorption band and an electrochemical LUMO energy or electron affinity of −3.07 eV was estimated from the onset of the first reduction waves (Table S2).

**FIGURE 4:**
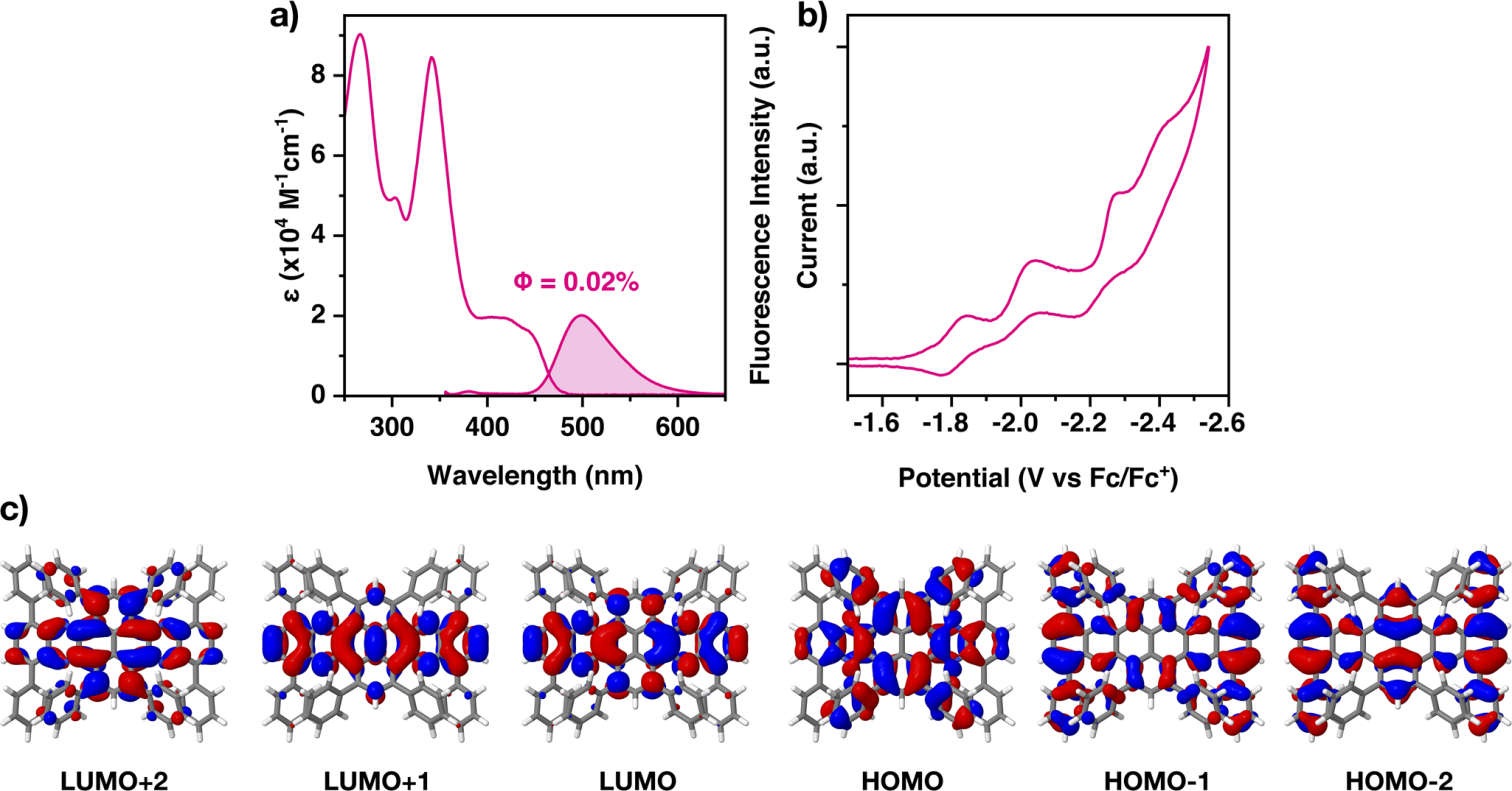
(a) Absorption and emission spectra of dibenzohexacene **1** in dichloromethane. (b) Cyclic voltammogram of dibenzohexacene **1** in a 0.1 M solution of *n*Bu_4_PF_6_ in dichloromethane. (c) Frontier orbitals computed with the B3LYP Hamiltonian with the 6‐31G(d,p) basis set in dichloromethane of *bent*‐**1**.

To shine light on the electronic properties of dibenzohexacene **1**, the frontier orbitals of the bent (Figure 4c and Table S3) and twisted (Figure S2 and Table S3) conformations were computed at the B3LYP/6‐311+G(2d,2p) level in dichloromethane. The energies and frontier orbitals are very similar in both cases, with the only notable change being an inversion of the HOMO and HOMO–1 energy levels (Figures 4b and S2). These subtle differences are, however, clearly reflected in the TD‐DFT‐calculated absorption spectra (Figure S3 and Tables S4–S5). Interestingly, molecular conformation strongly affects the computed TD spectra, with the weakly populated *twisted*‐**1** conformer exhibiting substantially enhanced absorption (Figure S3). The TD‐DFT absorption spectrum of conformer *bent*‐**1** shows a closer resemblance to the experimental one, consistent with the preferential population of the bent conformation in solution. TD‐DFT calculations revealed that the lowest‐energy excitation of the bent conformer is predominantly assigned to a HOMO→LUMO transition (Table S4). In contrast, the second excitation, which is the most intense, is mainly associated with a HOMO→LUMO+1 transition, whereas the third excitation is predominantly associated with a HOMO–1→LUMO transition.

In summary, we have described a pyrene‐fused azaacene (**1**) in which steric overcrowding, introduced by eight strategically positioned phenyl substituents, enforces bending of the extended aromatic framework rather than twisting. This sterically induced deformation results in a negatively curved, saddle‐like geometry with pronounced deviations from planarity, as evidenced by bend angles of 41° and 34° determined by single‐crystal x‐ray diffraction. While bending is primarily driven by steric effects, intramolecular π‐π stacking and an efficient π‐π overlap contribute to the stabilization of the bent conformation. Overall, this work provides a new approach to the synthesis of bent polycyclic aromatic hydrocarbons and molecular nanographenes. Extending this approach to elongated π‐conjugated systems may offer a means to modulate graphene nanoribbon curvature and topology at the molecular level, thereby enabling controlled access to wavy or belt‐like architectures and further expanding the synthetic toolbox for carbon nanomaterials.

## Author Contributions

**Marco Carini**: investigation, writing – review and editing.**Miguel Martín‐Arroyo**: Investigation, Writing ‐ review & editing. **Manuel Melle‐Franco**: Investigation, Writing ‐ review & editing. **Aurelio Mateo‐Alonso**: Conceptualization, Investigation, Writing ‐ original draft.

## Conflicts of Interest

The authors declare no conflict of interest.

## Supporting information




**Supporting File 1**: The authors have cited additional references within the Supporting Information [73].

## Data Availability

The data that support the findings of this study are available in the supplementary material of this article.
