# Supplementary material for: A Negatively Curved Pyrene‐Fused Azaacene
Source: Angew Chem Int Ed Engl. 2026 Apr 29;65(24):e3918437. doi: 10.1002/anie.3918437 (PMC13245604; doi:10.1002/anie.3918437)
Supplement: Supplementary file 1 — Supporting File 1: The authors have cited additional references within the Supporting Information [73]. [file ANIE-65-e3918437-s001.pdf]

A Negatively Curved Pyrene-Fused Azaacene

Marco Carini,[a] Miguel Martín-Arroyo,[a] Manuel Melle-Franco[b] and Aurelio Mateo-Alonso[a,c]*

[a] *POLYMAT, Department of Applied Chemistry, University of the Basque Country (EHU), 20018 Donostia-San Sebastián, Spain.*

E-mail: amateo@polymat.eu

[b] *CICECO—Aveiro Institute of Materials, Department of Chemistry, University of Aveiro, 3810–193 Aveiro, Portugal*

[c] *Ikerbasque, Basque Foundation for Science, 48011 Bilbao, Spain.*

Table of contents

Supplementary Schemes	S3
Supplementary Figures	S4
Supplementary Tables	S7
General Methods	S12
Synthetic procedures	S14
NMR Spectra	S16
Cyclic voltammetry	S19
References	S20

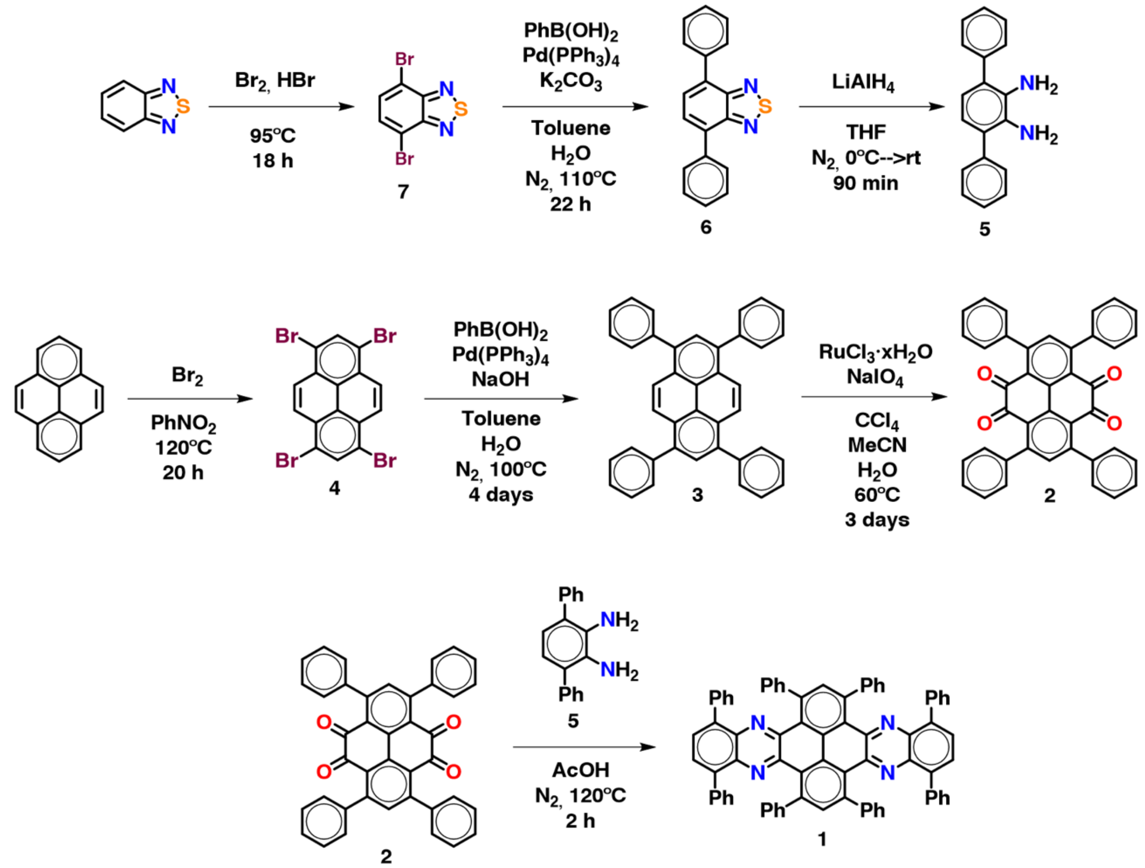

Scheme S1. Complete synthetic route for the synthesis of dibenzohexacene **1**.

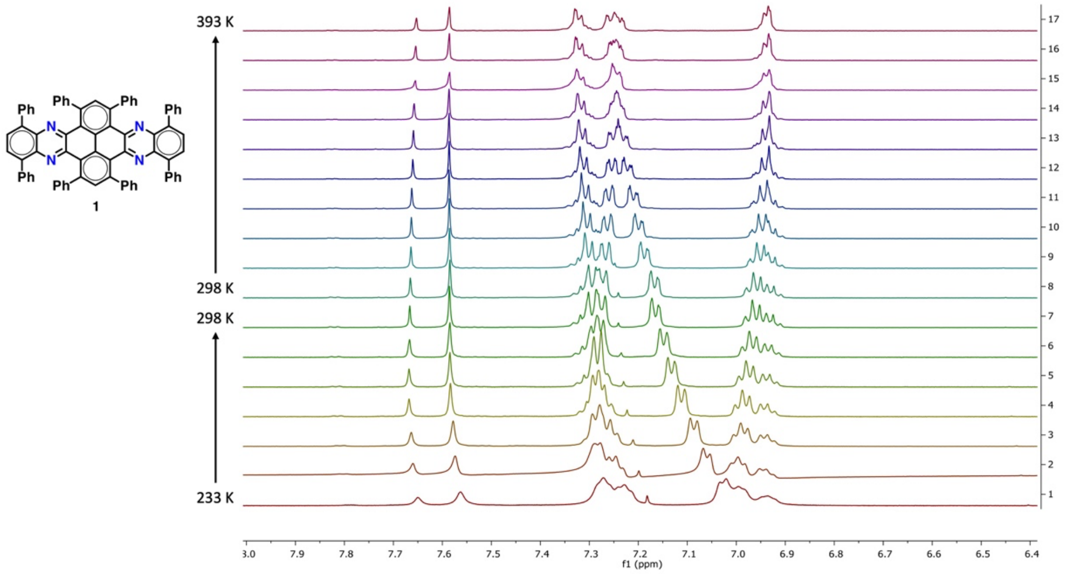

Figure S1. VT ^{1}H-NMR of **1**, TCE-d_2, 400 MHz.

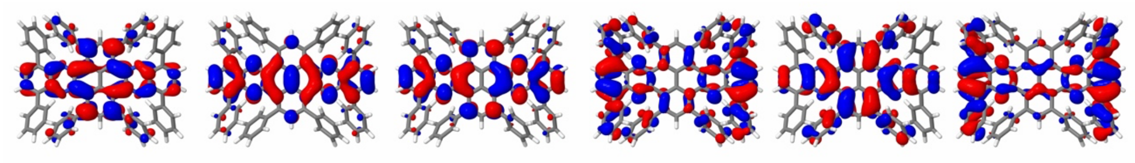

Figure S2. Frontier orbitals computed with the B3LYP Hamiltonian with the 6-31G(d,p) basis set in dichloromethane of *twisted*-**1**.

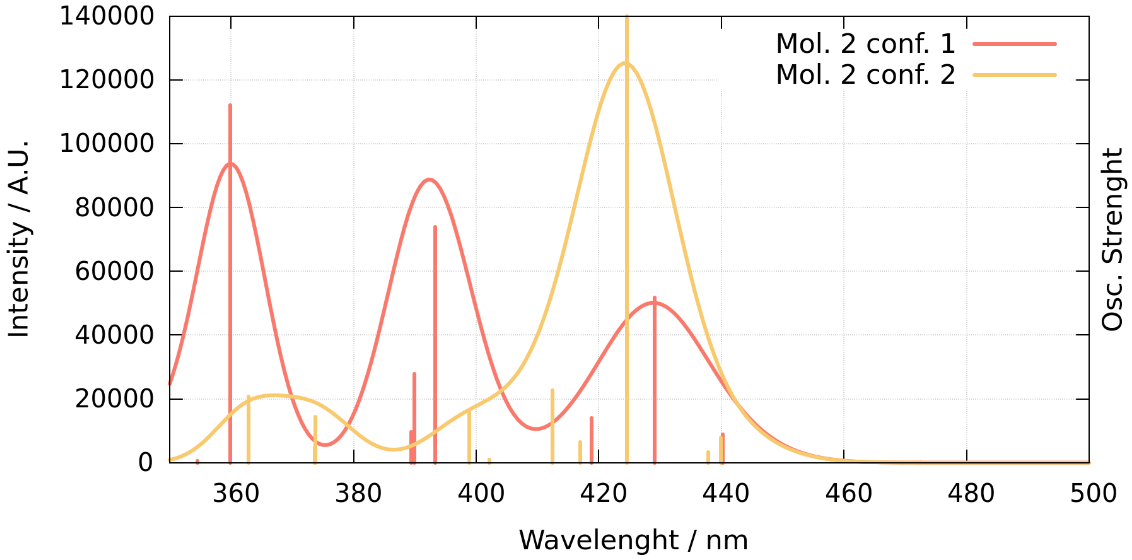

Figure S3. TD-DFT first 12 excitations computed with the B3LYP Hamiltonian with the 6-311+G(2d,p) basis set in dichloromethane of *bent*-**1** (red trace) and *twisted*-**1** (orange trace). All geometries were optimized with the ωB97X-3c Hamiltonian.

Table S1. Total energies, relative energies with respect to the global minimum, and populations at 25 C in gas-phase, with thermal corrections, and in dichloromethane computed with the ωB97X-3c Hamiltonian.

ωB97X-3c									
	**Gas Phase**			**Thermal Corr.**			**Dichloromethane**		
**Mol. - Conf. #**	**Total E. (eH)**	**Rel E. (kcal/mol)**	**Pop.**	**G (eH)**	**Rel G (kcal/mol)**	**Pop.**	**Total G (eH)**	**Rel E. (kcal/mol)**	**Pop.**
bent-**1**	-500.376055930	0.0	75.7%	-499.302892230	0.0	74.8%	-500.191372653	0.0	94.3%
twisted-**1**	-500.374461519	1.0	14.0%	-499.301359240	1.0	14.7%	-500.186492646	3.1	0.5%
bent-twisted-**1**	-500.373627829	1.5	5.8%	-499.300521270	1.5	6.1%	-500.186971539	2.8	0.9%
asymetrically-twisted-**1**	-500.372281734	2.4	1.4%	-499.298912590	2.5	1.1%	-500.188439628	1.8	4.2%
alternately-twisted-**1**	-500.373053044	1.9	3.1%	-499.299939000	1.9	3.3%	-500.184844133	4.1	0.1%

Table S2. The potentials are reported as $E_{1/2} = (E_{pa} + E_{pc})/2$ and rounded to the nearest 0.01 V. Optical band gap calculated using equation E_g = hc/λ_{ae} ≈ 1240/λ_{ae} (nm); Where λ_{ae} denotes the absorption edge wavelength in nm, obtained from onset wavelength derived from the lowest energy absorption band in dichloromethane. The LUMO levels were estimated from the onset of the first reduction potential $E_{LUMO} = -(E_{1red(ONSET)} + 4.8$ eV).

Compound	λ_{ons} (nm)	E_{gap} (eV)	E_{ons} (V)	E_{LUMO} (eV)	$E_{1/2}$ (V)	E_{red2} (V)	E_{red3} (V)	E_{red4} (V)
**1**	472	2.63	-1.73	-3.07	-1.81	-2.04	-2.29	-2.43

Table S3. Frontier orbitals computed with the B3LYP Hamiltonian with the 6-311+G(2d,p) basis set in dichloromethane of **1** in selected conformations. All geometries were optimized with the ωB97X-3c Hamiltonian. The colour code is: LUMOs, HOMOs and GAPs. **All values in eV**.

B3LYP-6-311+G(2d,p)-dichloromethane/ωB97X-3c								
**Mol. - Conf.**	LUMO+2	LUMO+1	**LUMO**	**HOMO**	HOMO-1	HOMO-2	HOMO-3	**Gap**
bent-**1**	-1.85	-2.36	**-2.48**	**-5.87**	-5.94	-5.98	-6.37	**3.39**
twisted-**1**	-1.9	-2.43	**-2.49**	**-5.88**	**-5.89**	-5.94	-6.15	**3.39**
bent-twisted-**1**	-1.9	-2.39	**-2.5**	**-5.86**	**-5.89**	-5.98	-6.22	**3.36**
asymetrically-twisted-**1**	-1.87	-2.35	**-2.49**	**-5.87**	-5.95	-6.02	-6.38	**3.38**
alternately-twisted-**1**	-1.86	-2.45	**-2.48**	**-5.88**	**-5.91**	-5.93	-6.25	**3.4**

Table S4. TD-DFT first 12 excitations computed with the B3LYP Hamiltonian with the 6-311+G(2d,p) basis set in dichloromethane of *bent*-**1**. All geometries were optimized with the ωB97X-3c Hamiltonian.

bent-**1**						
# exc	eV	nm	Osc. Strength	Contributions		
1	2.82	440	0.03	HOMO->LUMO(86%)	H-6->LUMO(5%)	H-3->L+1(7%)
2	2.89	429	0.20	H-3->LUMO(18%)	HOMO->L+1(74%)	H-6->L+1(4%)
3	2.96	419	0.05	H-2->L+1(16%)	H-1->LUMO(82%)	
4	2.99	415	0.00	H-2->LUMO(84%)	H-1->L+1(13%)	
5	3.12	398	0.00	H-4->LUMO(15%)	H-1->L+1(74%)	H-2->LUMO(9%)
6	3.15	393	0.28	H-6->L+1(19%)	H-3->LUMO(49%)	HOMO->L+1(22%)
7	3.18	390	0.11	H-2->L+1(77%)	H-1->LUMO(15%)	H-5->LUMO(2%)
8	3.18	389	0.04	H-6->LUMO(39%)	H-3->L+1(39%)	HOMO->LUMO(13%)
9	3.38	367	0.00	H-4->LUMO(83%)	H-1->L+1(12%)	H-2->LUMO(4%)
10	3.45	360	0.42	H-5->LUMO(29%)	HOMO->L+2(58%)	H-13->L+1(3%)
11	3.50	355	0.00	H-4->L+1(87%)	H-5->LUMO(3%)	H-2->L+1(3%)
12	3.55	349	0.04	H-5->LUMO(59%)	HOMO->L+2(33%)	

Table S5. TD-DFT first 12 excitations computed with the B3LYP Hamiltonian with the 6-311+G(2d,p) basis set in dichloromethane of *twisted*-**1**. All geometries were optimized with the ωB97X-3c Hamiltonian.

twisted-**1**						
# exc	eV	nm	Osc. Strength	Contributions		
1	2.82	440	0.03	H-3->L+1(13%)	H-2->L+1(15%)	HOMO->LUMO(57%)
2	2.83	438	0.01	H-3->LUMO(29%)	H-2->LUMO(20%)	HOMO->L+1(37%)
3	2.91	426	0.00	H-1->LUMO(97%)		
4	2.92	425	0.53	H-1->L+1(94%)	H-5->LUMO(2%)	
5	2.97	417	0.02	H-4->L+1(10%)	H-3->LUMO(32%)	H-2->LUMO(54%)
6	3.01	412	0.09	H-4->LUMO(24%)	H-3->L+1(41%)	H-2->L+1(18%)
7	3.08	402	0.00	H-3->LUMO(10%)	H-2->LUMO(24%)	HOMO->L+1(59%)
8	3.11	399	0.06	H-2->L+1(63%)	HOMO->LUMO(30%)	H-3->L+1(3%)
9	3.32	374	0.05	H-4->LUMO(47%)	H-3->L+1(37%)	H-6->L+1(7%)
10	3.32	374	0.02	H-6->LUMO(17%)	H-4->L+1(51%)	H-3->LUMO(26%)
11	3.42	363	0.08	H-5->LUMO(74%)	H-11->L+1(2%)	H-3->L+2(8%)
12	3.45	359	0.00	HOMO->L+2(78%)	H-11->LUMO(2%)	

General methods

Reagents. All the commercial reagents utilized in the synthesis were used as received. 1,3,6,8-tetraphenylpyrene (**4**)[1,2] and [1,1':4',1''-terphenyl]-2',3'-diamine (**5**)[3,4] were synthesized according to the literature.

Synthesis. All the reactions were performed in an oven-dried round-bottom flask, Schlenk tube or reaction vial. The required high temperature for the reactions was achieved using an oil-bath or an aluminum heating block, unless otherwise noted. The reaction progress was regularly monitored by thin-layer chromatography on TLC plates for which visualization was realized either by visual observation with naked eye or by irradiation with UV lamp.

Purification. The purification of the products was performed by silica-gel column chromatography under ambient conditions. The sorbent for the column chromatography (silica gel 60, 0.04–0.06 mm, 230–400 mesh) and the TLC plates were purchased from commercial suppliers. The monitoring of the purification process was performed by thin-layer chromatography on TLC plates.

Characterization. The NMR spectra were recorded with 400 or 500 MHz pulsed Fourier transform NMR spectrometer in deuterated solvents at room temperature. The chemical shifts are measured in ppm and *J* values in Hz, using the signals of the deuterated solvent as the internal standard [$CDCl_3$ calibrated at 7.26 ppm (^{1}H) and 77.2 ppm (^{13}C), TCE-d_2 calibrated at 6.00 ppm (^{1}H) and 73.8 ppm (^{13}C)]. High-resolution mass spectra of all the compounds were recorded by Dr. Javier Calvo on UltrafleXtreme III MALDI tandem mass spectrometer (Bruker) in reflector acquisition operation mode and the samples were prepared in THF.

Absorption and emission spectroscopy. The absorption spectra were recorded with a double beam UV/VIS/NIR spectrophotometer (PerkinElmer – Lambda 950) and the emission spectra were collected on a fluorescence spectrometer (PerkinElmer – LS 55).

Quantum Yield calculation. The fluorescence quantum yield was determined with by the use of a standard with the equation:

$$\Phi = \Phi_s * \frac{slope}{slope_s} * \frac{\eta^2}{\eta_s^2}$$

Where the slope is obtained representing the integrated emission intensity versus the absorbance. Compound **1** was dissolved in CH_2Cl_2 and the absorption and emission (λ_{exc}= 365 nm) were recorded. The representation of emission vs absorbance gave a value of 29315. The same procedure was repeated with acridine in ethanol, with a known quantum yield of 0.79%,[5] obtaining a value of 622727 when emission vs absorbance were represented. With these values, the fluorescence quantum yield of **1** was estimated to be 0.02%.

	QY	slope	r	refractive index
**Sample (DCM)**	0.02%	29315	0.9978	1.4241
**Acridine (EtOH)**	0.79%	622727	0.9946	1.3614

Exc. wavelength: 365 nm
Slit: 9

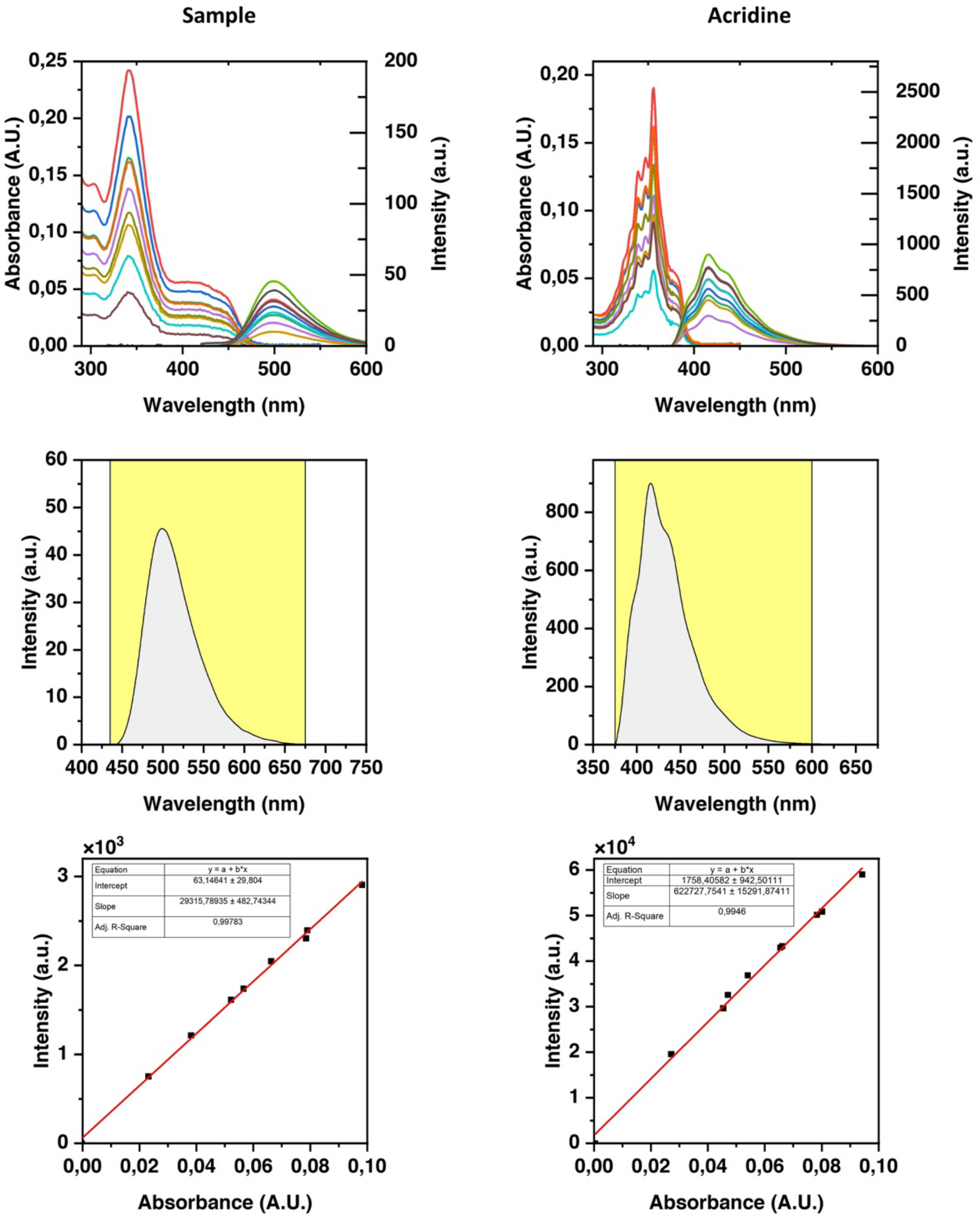

Figure S4. Fluorescence quantum yield estimation.

Synthetic procedures

Synthetic procedures

Synthesis of 1,3,6,8-tetraphenylpyrene-4,5,9,10-tetraone (2)

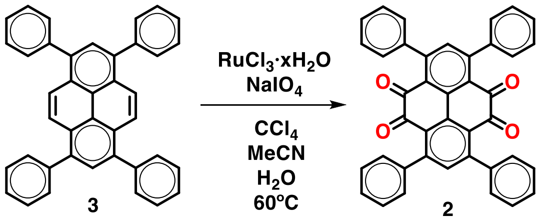

1.00 g of 1,3,6,8-tetraphenylpyrene (1.97 mmol) was dissolved in 30 ml of CCl_4 and 30 ml of MeCN, 12 mg of $RuCl_3$•xH_2O (0.197 mmol) were added with stirring followed by 37.5 ml of water. 3.80 g of $NaIO_4$ (17.8 mmol) were added in small portions with vigorous stirring. The reaction was stirred at 60°C for 3 days. The product was extracted with dichloromethane / water, the combined organic phase was dried over Na_2SO_4 and evaporated. The product was triturated with MeOH, filtered and washed with MeOH until the filtrate is colorless. 194 mg (0.343 mmol) were obtained as red powder in a 17% yield.

^{1}H NMR (400 MHz, $CDCl_3$) δ 7.44 – 7.40 (m, 12H), 7.39 (s, 2H), 7.31 (dd, *J* = 6.7, 2.9 Hz, 8H).
^{13}C NMR (101 MHz, $CDCl_3$) δ 185.53, 151.25, 139.15, 138.53, 137.33, 129.25, 128.76, 128.71, 128.09.
MS (MALDI-TOF, DCTB Na): Calculated for $C_{40}H_{22}O_4Na$ $[M+Na]^+$: 589.1414; Found: 589.1440.

Synthesis of Hexacene 1

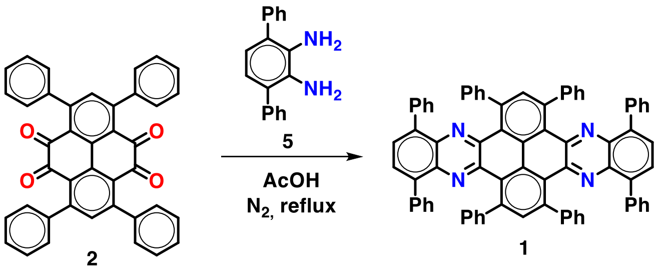

100 mg of **2** (0.177 mmol) and 138 mg of diamine **5** (0.530 mmol) were suspended in 3 ml of AcOH and the mixture was refluxed under N_2 atmosphere for 2 hours. The reaction was cooled to room temperature and diluted with MeOH, the precipitate was filtered and washed with MeOH. The product was purified by column chromatography in dichloromethane → $CHCl_3$ and it was finally precipitated from $CHCl_3$ to MeOH, filtered and washed with MeOH. The product was crystallized from hot $CHCl_3$ / EtOH. 124 mg (0.122 mmol) were obtained as yellow crystalline powder in a 69% yield.

^{1}H NMR (400 MHz, TCE-d_2) δ 7.71 (s, 2H), 7.62 (s, 4H), 7.41 – 7.28 (m, 20H), 7.25 – 7.19 (m, 8H), 7.05 – 6.93 (m, 12H).
^{13}C NMR (101 MHz, TCE-d_2) δ 143.73, 142.93, 142.56, 139.87, 138.67, 137.37, 133.02, 130.59, 129.10, 128.47, 128.37, 127.20, 126.79, 126.67.
MS (MALDI-TOF, DCTB Na): Calculated for $C_{76}H_{46}N_4Na$ $[M+Na]^+$: 1037.3616; Found: 1037.3574.

NMR Spectra

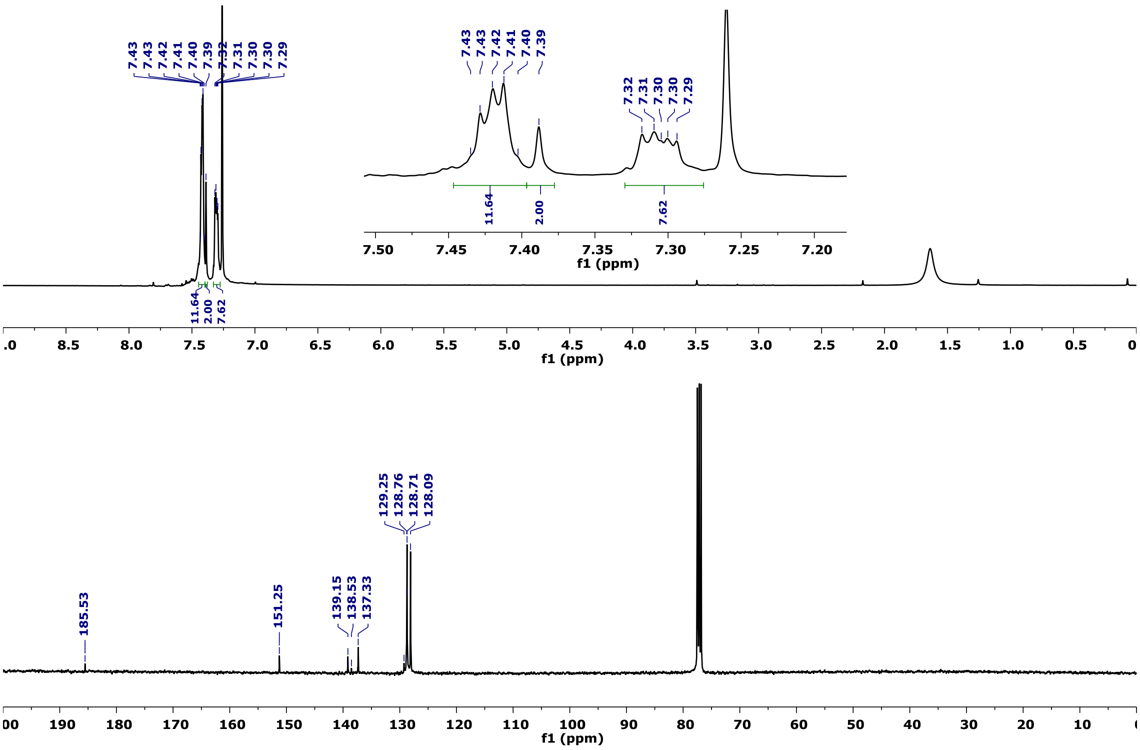

Figure S5. ^{1}H and ^{13}C NMR of **2**, $CDCl_3$ at 298 K, 400 MHz.

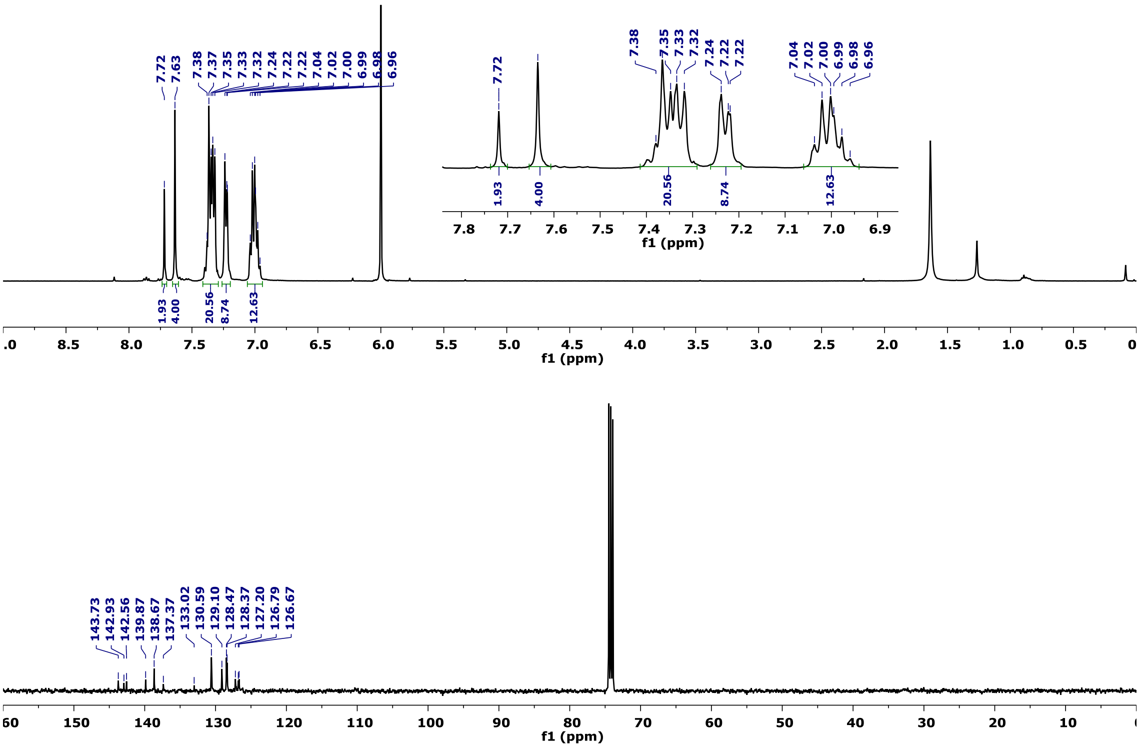

Figure S6. ^{1}H and ^{13}C NMR of **1**, TCE-d_2 at 298 K, 400 MHz.

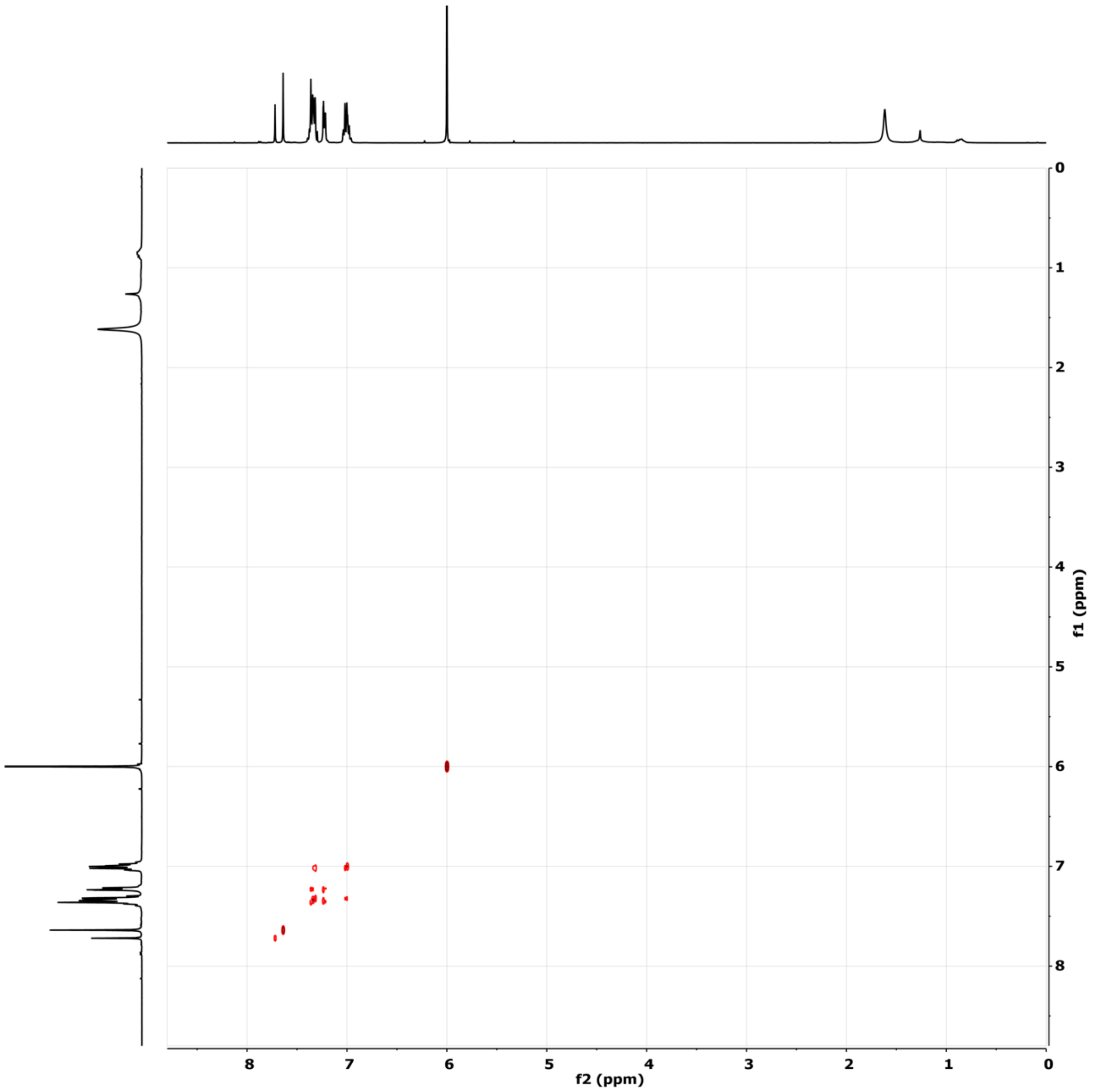

Figure S7. COSY of **1**, TCE-d_2 at 298 K, 400 MHz.

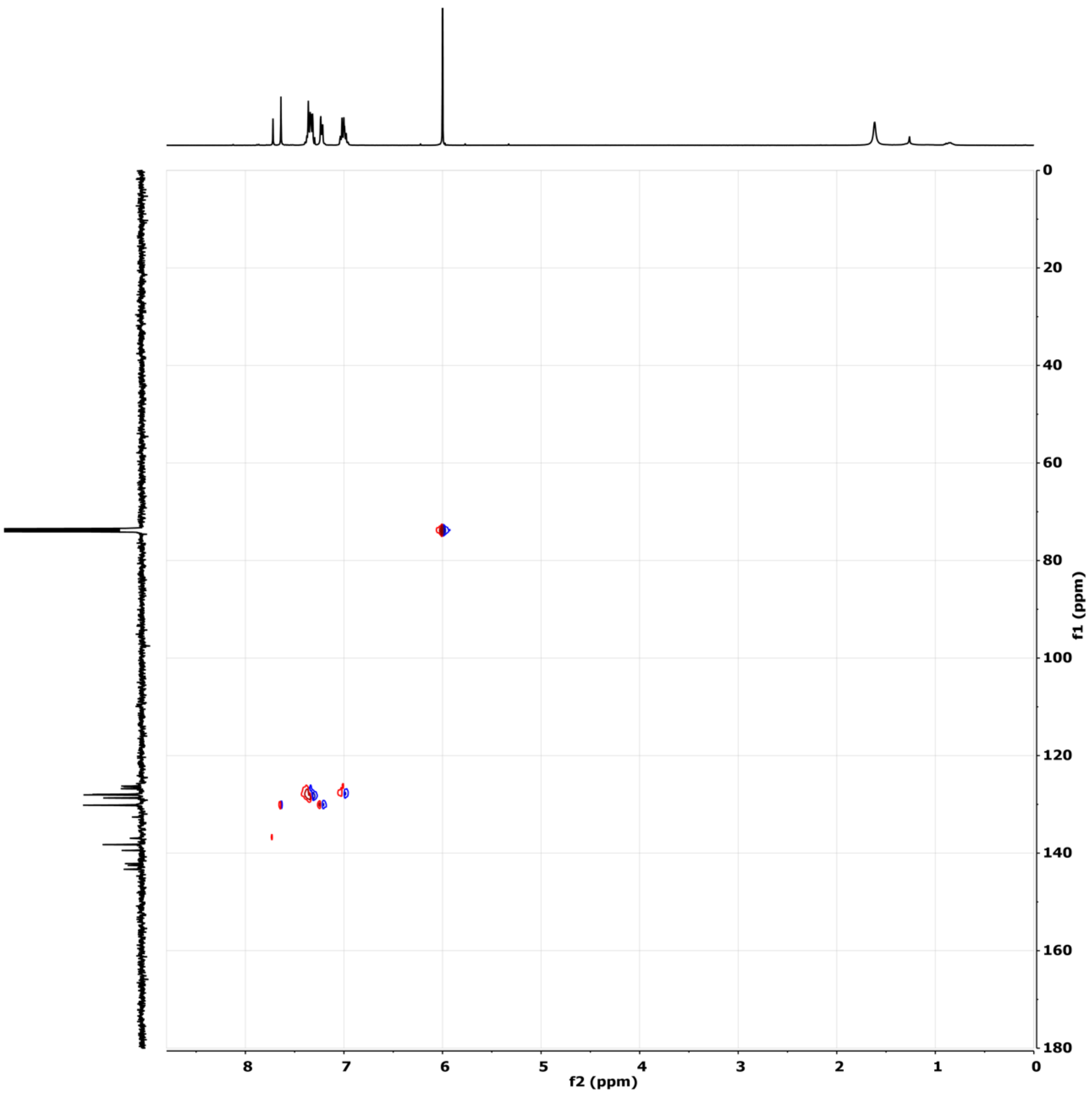

Figure S8. HSQC of **1**, TCE-d_2 at 298 K, 400 MHz

Cyclic voltammetry

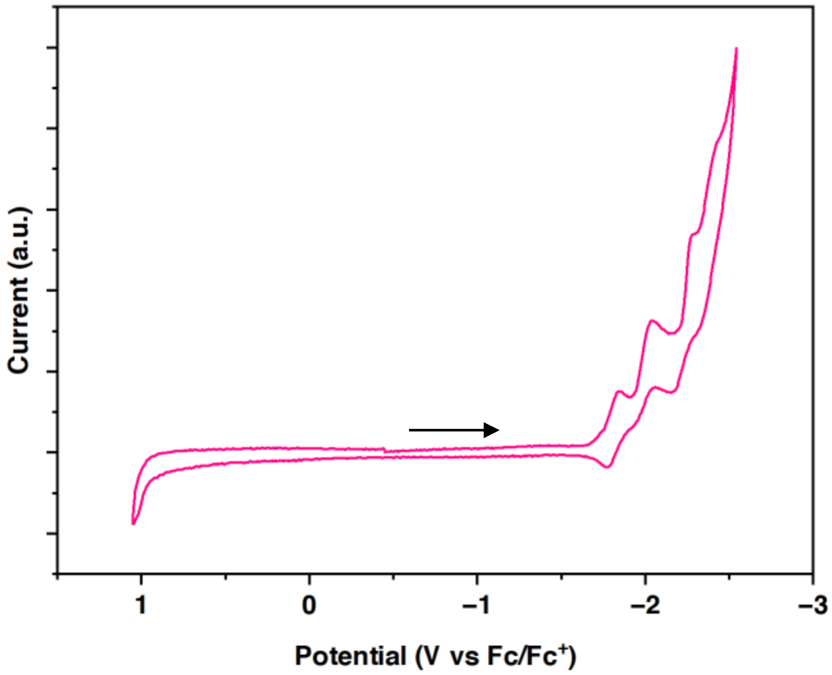

Figure S9. Cyclic voltammogram of **1** (0.26 mM) in CH_2Cl_2 (0.1M nBu_4NPF_6). Scan rate = 125 mVs^{-1}, working electrode: GC, counter electrode: Pt, reference electrode: Ag.

References

[1] G. Venkataramana, S. Sankararaman, *Eur. J. Org. Chem.* **2005**, 4162–4166.

[2] X. Feng, H. Tomiyasu, J.-Y. Hu, X. Wei, C. Redshaw, M. R. J. Elsegood, L. Horsburgh, S. J. Teat, T. Yamato, *J. Org. Chem.* **2015**, *80*, 10973–10978.

[3] S. Choudhary, C. Gozalvez, A. Higelin, I. Krossing, M. Melle-Franco, A. Mateo-Alonso, *Chem. Eur. J.* **2014**, *20*, 1525–1528.

[4] T. Shigehiro, S. Yagi, T. Maeda, H. Nakazumi, H. Fujiwara, Y. Sakurai, *Tetrahedron Lett.* **2014**, *55*, 5195–5198.

[5] M. Taniguchi, J. S. Lindsey, *Photochem. Photobiol.* **2018**, *94,* 290–327.
